# Characterization and Online Detection of Surfactin Isomers Based on HPLC-MS^n^ Analyses and Their Inhibitory Effects on the Overproduction of Nitric Oxide and the Release of TNF-α and IL-6 in LPS-Induced Macrophages

**DOI:** 10.3390/md8102605

**Published:** 2010-10-11

**Authors:** Jin-Shan Tang, Feng Zhao, Hao Gao, Yi Dai, Zhi-Hong Yao, Kui Hong, Jia Li, Wen-Cai Ye, Xin-Sheng Yao

**Affiliations:** 1 Institute of Traditional Chinese Medicine & Natural Products, Jinan University, Guangzhou 510632, Guangdong, China; E-Mails: gztangjinshan@126.com (J.-S.T.); daiyi1004@163.com (Y.D.); tyaozh@jnu.edu.cn (Z.-H.Y.); chywc@yahoo.com.cn (W.-C.Y.); 2 School of Pharmacy, Yantai University, Yantai 206005, Shandong, China; E-Mail: zhaofeng@ytu.edu.cn; 3 State Key Laboratory of Drug Research, Shanghai Institute of Materia Medica, Shanghai 201203, China; E-Mail: tghao@jnu.edu.cn; 4 Institute of Tropical Biosciences and Biotechnology, Chinese Academy of Tropical Agriculture Sciences, Haikou 571101, Hainan, China; E-Mail: K1102@163.net; 5 National Center for Drug Screening, Shanghai Institute of Materia Medica, Shanghai 201203, China; E-Mail: jli@mail.shcnc.ac.cn

**Keywords:** LC-MS, Bacillus sp., surfactin isomer, anti-inflammatory activity

## Abstract

A rapid method for characterization and online detection of surfactin isomers was developed based on HPLC-MS^n^ (n = 1, 2, 3) analyses, and many surfactin isomers were detected and characterized from the bioactive fraction of the mangrove bacterium *Bacillus* sp. Inhibitory activities of surfactin isomers on the overproduction of nitric oxide and the release of TNF-α and IL-6 in LPS-induced macrophages were systematically investigated. It was revealed that the surfactin isomers showed strong inhibitory properties on the overproduction of nitric oxide and the release of IL-6 on LPS-induced murine macrophage cell RAW264.7 with IC_50_ values ranging from 1.0 to 7.0 μM. Structure-activity relationship (SAR) studies revealed that the existence of the free carboxyl group in the structure of surfactin isomers was crucial. These findings will be very helpful for the development of this novel kind of natural product as new anti-inflammatory agents.

## 1. Introduction

**Figure 1:**
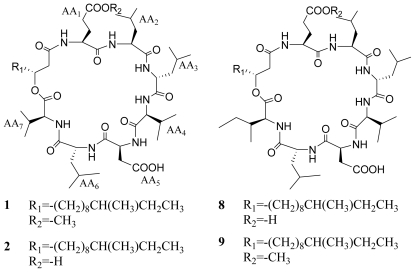

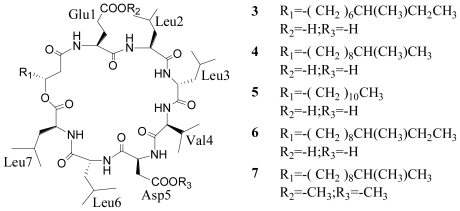
Chemical structures of compounds **1**–**9** obtained from the bacterium *Bacillus* sp.

Surfactin isomers are cyclic lipopeptide biosurfactants consisting of seven amino acid units and one β-hydroxyl fatty acid side chain with diverse chain lengths of 13–15 carbons which are characteristic metabolites of the genus *Bacillus.* Surfactin isomers have received much attention during the last two decades since they exhibit numerous pharmaceutical activities including anticoagulation [1], anti-tumor [2], antiviral [3], anti-inflammatory, and immunosuppressive activities [4–7]. Surfactin isomers are best known for their multifaceted interactions with biological systems that result in a number of physiological and biochemical activities [8], and can incorporate into the phospholipid bilayer and induce permeabilization and perturbation of target cell owing to their amphipathic nature. These characteristics make them promising for the treatment of a number of global public health issues. High performance liquid chromatography coupled with tandem mass spectrometry (HPLC-MS^n^) is one of the most powerful techniques for online analysis of complex components in a crude extract. A variety of natural products, such as flavonoids, alkaloids, saponins, and steroids [9–12], have been analyzed by HPLC-MS^n^. During our search for bioactive metabolites from marine microorganisms, a series of surfactin isomers was obtained from the bacterium *Bacillus* sp. (Figure 1) [13]. In this paper, we developed a fast and reliable method for characterizing trace amounts of surfactin isomers from the bioactive fraction (061341-A9) of the mangrove bacterium *Bacillus* sp. based on rules deduced from the relationship between the fragmentation behaviors and characteristic structure features. At the same time, inhibitory activities of surfactin isomers on the overproduction of nitric oxide and the release of TNF-α and IL-6 in LPS-induced macrophages were simultaneously investigated.

## 2. Results and Discussion

### 2.1. Fragmentation behavior of pure surfactin isomers (1–9)

The fragmentation behavior of nine pure surfactin isomers was investigated by ESI-MS^n^ (n = 1, 2, 3) experiments, which indicated that they shared similar fragmentation routes. The full-scan mass spectra showed intense pseudo-molecular ions [M + H]^+^ at *m/z* 1036 (**1**, **6**, **8**), 1022 (**2**, **4**, **5**), 1008 (**3**), and 1050 (**7**, **9**) in the positive ion mode and showed intense pseudo-molecular ions [M − H]^−^ at *m/z* 1034 (**1**, **6**, **8**), 1020 (**2**, **4**, **5**), 1006 (**3**), and 1048 (**7**, **9**) in the negative ion mode, respectively (Table 1). The MS^2^ spectra of precursor ion [M + H]^+^ were dominated by a common ion peak at *m/z* 671 (**1**, **2**), 685 (**3**–**6**, **8**–**9**), and 699 for **7**, respectively, which was attributed to the product ion [(H) AA_2_ − AA_7_ (OH) + H]^+^. The presence of this ion indicated the preferential opening of the ring at the ester site, which was consistent with a previous report [14]. In the MS^2^ spectra of precursor ion [M + H]^+^, the neutral loss of AA_7_ + H_2_O [117 Dalton (Val + H_2_O) for **1** and **2**; 131 Dalton (Leu or Ile + H_2_O) for **3**–**9**] was also observed, which derived from a double hydrogen transfer (**DHT**) of the ester bond of the cyclic skeleton and cleavage of one *C*-terminal amino acid residue. The presence of this ion could be used to identify the *C*-terminal amino acid (AA_7_) without acid hydrolysis [15]. In the MS^3^ spectra of precursor ion [(H) AA_2_ − AA_7_ (OH) + H]^+^, the neutral loss of AA_7_ + H_2_O and AA_7_ + AA_6_ + H_2_O were also predominant, which further confirmed the kind of *C*-terminal amino acid. Pseudo-molecular ions and main product ions of pure surfactin isomers (**1**–**9**) are displayed in Table1. Previous biogenesis research revealed that the kinds of amino acid residues AA_1_ (Glu), AA_3_ (Leu), AA_5_ (Asp), and AA_6_ (Leu) of surfactin isomers were usually conservative and those of amino acid residues AA_2_ (Leu/Ile), AA_4_ (Val/Leu) and AA_7_ (Val/Leu/Ile) were replaceable [8]. So, it was not difficult to characterize the structure of surfactin isomers according to MS/MS spectral data.

### 2.2. On-line characterization of surfactin isomers by HPLC-MS^n^ (n = 1, 2, 3)

**Figure 2:**
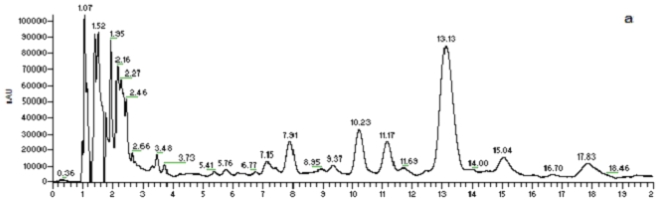

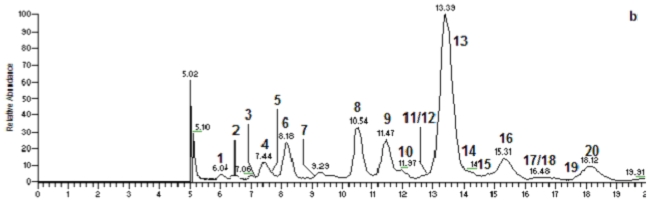
HPLC-UV-ESI-ITMS analysis of the fraction (061341-A9) derived from the mangrove bacterium *Bacillus* sp. (**a**): UV chromatogram at 220 nm; (**b**): total ion chromatogram (TIC) in the positive ion mode.

**Figure 3:**
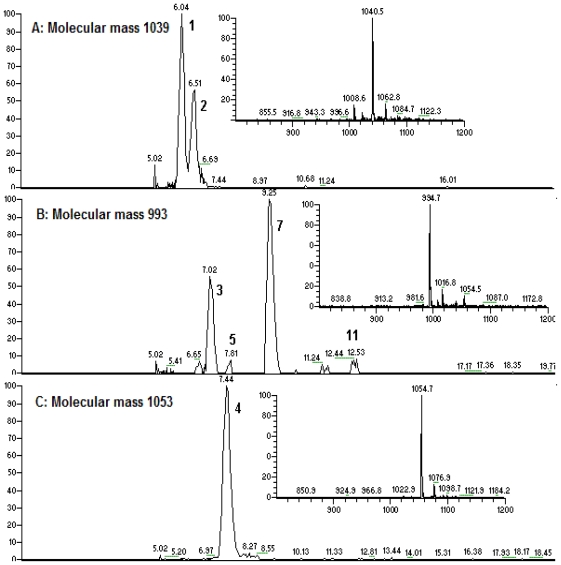

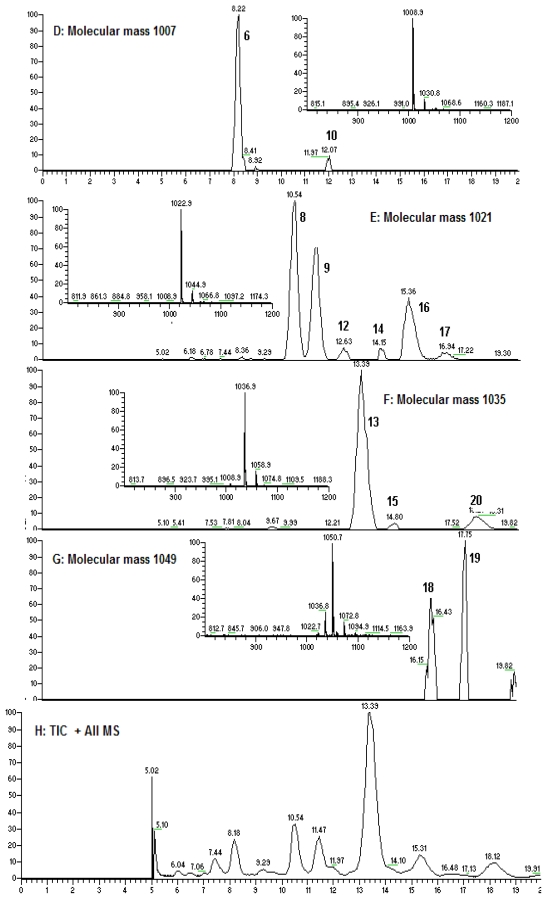
(A–G): Extracted ion chromatogram for *m/z* 1040 (**A**), 994 (**B**), 1054 (**C**), 1008 (**D**), 1022 (**E**), 1036 (**F**), and 1050 (**G**); (**H**): TIC for the fraction (061341-9A).

A rapid method for characterizing surfactin isomers from the fraction (061341-A9) of the mangrove bacterium *Bacillus* sp. was developed based on HPLC-MS^n^ (n = 1, 2, 3) analyses. Initially, when only MeOH-H_2_O or ACN-H_2_O solvent systems were used as mobile phase, no peak was observed. To obtain better separation and more peaks, a mobile phase of 90% MeOH/H_2_O (0.05% CF_3_COOH) was adopted. 0.05% CF_3_COOH in the mobile phase could suppress the dissociation of the free carboxyl group in the structure of surfactin isomers. Figure 2 displays the HPLC fingerprint map and total ion chromatogram (TIC) of the fraction 061341-A9. Twenty peaks were detected from it and the corresponding peak numbers, retention times, pseudo-molecular ions, and main product ions of them are displayed in Table 2 (Figure 3). Peaks at 8.22 (peak 6), 10.54 (peak 8), 11.51 (peak 9), 13.39 (peak 13), 15.36 (peak 16), and 18.21 min (peak 20) were unambiguously attributed to compounds **2**–**6**, and **8**, respectively, by comparing the retention times and mass spectra with reference standards obtained from the mangrove bacterium *Bacillus* sp. (No. 061341) [13]. Apart from the six compounds mentioned above (**2**–**6**, **8**), some trace amounts of surfactin isomers were also detected from the fraction (061341-A9). Based on the rules deduced from the fragmentation behavior of pure surfactin isomers (**1**–**9**), eleven surfactin isomers were characterized based on HPLC-MS^n^ (n = 1, 2, 3) analyses, which could be classified into three groups according to their chemical structures.

#### 2.2.1. Group 1, linear derivatives of surfactin isomer

**Figure 4:**
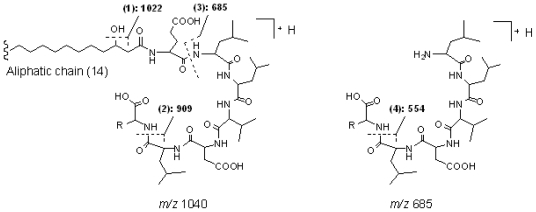
The main fragmentation routes of peak 1 (group 1, linear derivative of surfactin isomer).

Peak 1 gave a pseudo-molecular ion at *m/z* 1040 [M + H]^+^, which indicated that its molecular weight was 1039. In the MS^2^ spectrum of [M + H]^+^ at *m/z* 1040, product ions at *m/z* 1022 [M − 18 + H]^+^, 909 [M − (AA_7_ + 18) + H]^+^, and 685 [(H) AA_2_ − AA_7_ (OH) + H]^+^ were observed, which suggested that it was a linear lipopeptide with a fatty acid side chain of 14 carbons, and AA_7_ was Leu or Ile. In the MS^3^ spectrum of precursor ion at *m/z* 685, product ions at *m/z* 554 [(H) AA_2_ − AA_7_ (OH) − 131 + H]^+^ and *m/z* 441 [(H) AA_2_ − AA_7_ (OH) − 131 − 113 + H]^+^ further confirmed the deduction mentioned above. The main fragmentation routes of peak 1 are displayed as Figure 4 (Group 1, linear derivative of surfactin isomer). Consequently, peak 2 and peak 4 were deduced to be linear lipopeptides with AA_7_ of Leu or Ile. The chain length of fatty acid side chains were 14 and 15 carbons, respectively.

#### 2.2.2. Group 2, surfactin isomers with AA_7_ of Leu or Ile

**Figure 5:**
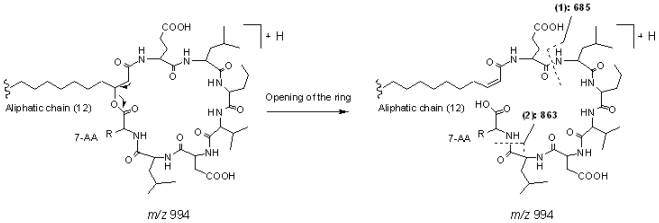
The main fragmentation routes of peak 3 (group 2, surfactin isomer with AA_7_ of Leu or Ile).

Peak 3 gave a pseudo-molecular ions at *m/z* 994 [M+H]^+^, which indicated that its molecular weight was 993. In the MS^2^ spectrum of [M + H]^+^, product ions at *m/z* 976 [M − 18 + H]^+^, 863 [M − (AA_7_ + 18) + H]^+^, and 685 [(H) AA_2_ − AA_7_ (OH) + H]^+^ were observed, which suggested that it was a cyclic lipopeptide with a fatty acid side chain of 12 carbons, and AA_7_ was Leu or Ile. The main fragmentation routes of peak 3 are displayed as Figure 5 (Group 2, surfactin isomer with AA_7_ of Leu or Ile). Consequently, peaks 14, 15, 18, and 19 were deduced to be cyclic lipopeptides with AA_7_ of Leu or Ile. The chain length of fatty acid side chains were 14, 15, 16, and 16, respectively.

#### 2.2.3. Group 3, surfactin isomers with AA_7_ of Val

**Figure 6:**
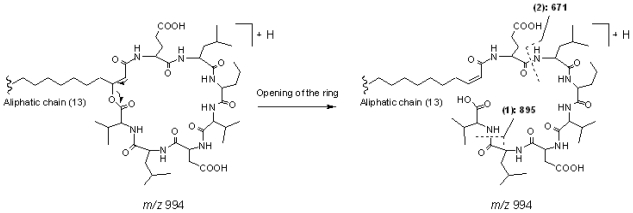
The main fragmentation routes of peaks 7 (Group 3, surfactin isomer with AA_7_ of Val).

Peak 7 gave pseudo-molecular ions at *m/z* 994 [M + H]^+^, which indicated that its molecular weight was 993. In the MS^2^ spectrum of [M + H]^+^, product ions at *m/z* 976 [M − 18 + H]^+^, 895 [M − (AA_7_ + 18) + H]^+^, and 671 [(H) AA_2_ − AA_7_ (OH) + H]^+^ were observed, which suggested that it was a cyclic lipopeptide with a fatty acid side chain of 13 carbons, and the kind of AA_7_ was Val. In the MS^3^ spectrum of precursor ion at *m/z* 671 [(H) AA_2_ − AA_7_ (OH) + H]^+^, product ions at *m/z* 653 [(H) AA_2_ − AA_7_ (OH) − 18 + H]^+^, 554 [(H) AA_2_ − AA_7_ (OH) − 117 + H]^+^, and 441 [(H) AA_2_ − AA_7_ (OH) − 117 − 113 + H]^+^ further confirmed the deduction mentioned above. The main fragmentation routes of peak 7 are displayed as Figure 6 (Group 3, surfactin isomer with AA_7_ of Val). Consequently, peaks 10 and 12 were deduced to be cyclic lipopeptides with AA_7_ of Val. The length of the fatty acid side chains were 14 and 15, respectively.

### 2.3. Anti-inflammatory properties of pure surfactin isomers (1–9) on LPS-induced murine RAW264.7

The effect of pure surfactin isomers on cell viability was examined by MTT method. MTT experimental results revealed that **1**–**6**, **8**, and **9** showed no cytotoxicity below 10 μM, but showed strong cytotoxicity at a concentration of 30 μM. **7** exhibited no cytotoxicity at a concentration of 30 μM (data not shown).

Anti-inflammatory experimental results showed that compounds **1**–**6**, **8**, and **9** exhibited strong inhibitory properties on the overproduction of nitric oxide on LPS-induced murine macrophage cell RAW264.7 with IC_50_ values ranging from 1.0 to 5.0 μM (Table 3). Meanwhile, effects of compounds **1**–**9** on the release of TNF-α and IL-6 were also investigated on LPS-induced murine macrophage cell RAW264.7, and experiments were conducted in the same way as it was in case of NO production inhibition and the same type of statistical evaluation was applied to the obtained data. Experimental results showed that compounds **1**–**6**, **8**, and **9** exhibited strong inhibitory properties on the release of IL-6 on LPS-induced murine macrophage cell RAW264.7 with IC_50_ values ranging from 1.0–7.0 μM (Table 4). They showed IC_50_ values ranging from 10 to 30 μM on the release of TNF-α on LPS-induced murine macrophage cell RAW264.7 (Table 4). Compound **7** showed no inhibitory property on the overproduction of nitric oxide and the release of IL-6 and TNF-α on LPS-induced murine macrophage cell RAW264.7 at a concentration of 30 μM.

Structure-activity relationship (SAR) studies revealed that the existence of the free carboxyl group was essential in the structure of surfactin isomer, since monomethylated derivatization of surfactin isomer decreased the inhibitory effects on the overproduction of nitric oxide and the release of IL-6 and TNF-α on LPS-induced murine macrophage cell RAW264.7. Dimethylated derivative ones showed no inhibitory effect on the overproduction of nitric oxide and the release of IL-6 and TNF-α on LPS-induced murine macrophage cell RAW264.7. In addition, the kind of AA_7_ in the moiety also affected the inhibitory effect of surfactin isomer on the overproduction of nitric oxide and the release of IL-6 and TNF-α on LPS-induced murine macrophage cell RAW264.7. 7-Val substitution in the moiety showed stronger activities than those of Leu and Ile substitutions. Furthermore, the chain length and substitution type of the branching methyl in the fatty acid chain also affected the inhibitory properties of surfactin isomer on the overproduction of nitric oxide and the release of IL-6 and TNF-α on LPS-induced murine macrophage cell RAW264.7. Chain lengths of 14 and 15 carbons showed stronger activities than that of 13 carbons.

## 3. Experimental Section

### 3.1. Materials

Pure surfactin isomers (**1**–**9**) were obtained from the mangrove bacterium *Bacillus* sp. (No. 061341) [13]. The purities were above 98% as determined by HPLC/UV analysis. Mouse monocyte-macrophage RAW264.7 (ATCC TIB-71) was purchased from the Chinese Academy of Science. RPMI 1640 medium, penicillin, streptomycin and fetal bovine serum were purchased from Invitrogen (NY, USA). Lipopolysaccharide (LPS), hydrocortisone, DMSO and MTT were obtained from Sigma. Mouse TNF-α ELISA kit and mouse IL-6 ELISA kit were purchased from R&D. Other chemical reagents were of HPLC grade.

### 3.2. ESI-MS^n^ (n = 1, 2, 3) analyses of pure surfactin isomers (1–9)

ESI-MS^n^ experiments were conducted using a LCQ Advantage ion trap mass spectrometer (ThermoFinnigan, USA) equipped with ESI ion resource and Xcalibur workstation software. The positive ion ESI conditions were as follows: capillary temperature, 220 °C; capillary voltage, 46 V; spray voltage, 4.6 kV; tube lens offset, 55 V; sheath gas (N_2_) flow rate, 25 arb; auxiliary gas (N_2_) flow rate, 10 arb. The negative ion ESI conditions were as follows: capillary voltage, −46 V; tube lens offset −55 V; the other parameters were the same as those of positive ion mode. The ESI-MS^n^ (n = 1, 2, 3) spectra were conducted in the presence of helium collision gas with relative collision energies varying from 25% to 30%. The sample solutions were introduced via a syringe pump at a flow rate of 125 μL min^−1^.

### 3.3. Preparation of the fraction contained surfactin isomers

*Bacillus* sp. (No. 061341) was isolated from soil collected from the Wenchang mangrove, Hainan, China. A voucher specimen has been deposited in the Institute of Tropical Biosciences and Biotechnology, Haikou, China. The cultivation (70 L) were lyophilized and macerated with acetone overnight four times and then filtered. The filtrate was concentrated to dryness under vacuum and yielded 1000 g crude extract, which was directly chromatographed over an open silica gel column (200–300 mesh), eluted with CHCl_3_-MeOH (100:0–60:40) to yield 13 fractions (A1–A13). Pharmacological experiments revealed that fraction A9 (061341-A9, 0.96%) showed strong anti-inflammatory activities on LPS-induced murine RAW264.7, which also exhibited cytotoxic activity against HepG 2 cell line. TLC experiments suggested that it contained mainly cyclic peptides, since it gave negative reaction with ninhydrin but was positive after hydrolyzation with concentrated HCl (6*N*).

### 3.4. HPLC-MS^n^ (n = 1, 2, 3) analysis of the fraction contained surfactin isomers

HPLC-MS^n^ experiments were conducted using a Finnigan LCQ HPLC-MS^n^ system (ThermoFinnigan, USA) equipped with a P2000 LC pump, an AS3000 autosampler, and a LCQ Advantage MAX ion trap tandem mass spectrometer with electrospray ionization (ESI) interface. Chromatographic separation was achieved on a reverse-phase (RP) C_18_ column (5 μm, 4.6 × 150 mm, COSMOSIL) at room temperature. Optimized separation condition was adopted using a mobile phase of 90% MeOH-H_2_O (0.05% CF_3_COOH) in order to obtain better separation of all the peaks. The mobile phase flow rate was 1.0 mL/min. the HPLC system was directly connected to ion trap (IT) mass spectrometer via electrospray ionization (ESI) interface with the stream splitting ratio at 4:1.

### 3.5. Anti-inflammatory activities of the pure surfactin isomers (1–9)

#### 3.5.1. Cell culture

RAW264.7 cells were incubated in RPMI 1640 medium supplemented with penicillin (100 U/mL), streptomycin (100 μg/mL) and 10% heat inactivated fetal bovine serum at 37 °C in a humidified incubator with 5% CO_2_.

#### 3.5.2. Cell viability assay

Cells in the exponential growth phase were seeded in a 96-well plate at a density of 5 × 10^5^ cell/mL. Samples (**1**–**9**) were added at indicated concentrations, respectively. Blank control received an equal amount of DMSO, which resulted in a final concentration of 0.2% DMSO in the culture medium. The mitochondrial-dependent reduction of MTT to formazan was used to measure cell respiration as an indicator of cell viability. Briefly, after 24 h incubation with or without sample (**1**–**9**, 1–30 μM), a MTT solution (final concentration was 200 μg/mL) was added and the cells were incubated for another 4 h at 37 °C. After removing the supernatant, 100 μL of DMSO was added to the cells to dissolve the formazan. The absorbance of each group was measured by using a microplate reader at a wavelength of 570 nm. The blank control consisted of untreated-cells was considered as 100% of viable cells. Results were expressed as percentage of viable cells when compared with blank control.

#### 3.5.3. Nitric oxide analysis

Nitric oxide was determined by measuring the amount of nitrite in the cell culture supernatant, using Griess reagent (mixture of equal volume of reagent A and reagent B, (A): 1% sulphanilamide; (B): 0.1% naphthylethylene diamine dihydrochloride in 10% H_3_PO_4_). Hydrocortisone was used as positive control. RAW264.7 cells were treated by lipopolysaccharides (LPS, 1 μg/mL) with or without samples (**1**–**9**, 1–30 μM) for 24 h, then briefly centrifuged. 100 μL of the cell culture supernatant were mixed with 100 μL of Griess reagent, followed by incubation for 10 min at room temperature. The absorbance at 540 nm was measured and the inhibitory rates were calculated by using a standard calibration curve prepared from different concentrations of sodium nitrite.

#### 3.5.4. Measurement of cytokines

Cells were treated by LPS (1 μg/mL) with or without samples (**1**–**9**, 1–30 μM) for 6 h. 100 μL of the culture supernatant was taken out to determine the level of TNF-α and IL-6 using respective enzyme-linked immunosorbent assay kit according to the manufacturer’s recommendations. Hydrocortisone was used as positive control.

## 4. Conclusions

A rapid method for characterizing surfactin isomers was successfully developed based on HPLC-MS^n^ analyses and many trace amounts were detected from the fraction (061341-A9) of the mangrove bacterium *Bacillus* sp. It was worth noting that three methylated surfactin isomer derivatives were not detected from it, which presumably were artifacts derived from addition of the solvent to the cyclic lipopeptide in the process of purification. Unfortunately, it was difficult to discriminate the Leu unit and Ile unit due to the same molecular weight. At the same time, the substitution type of the branching methyl in the fatty acid chain was also unidentified only based on MS/MS information.

Pharmacological experimental results further confirmed that this class of cyclic lipopeptide showed strong inhibitory properties on the overproduction of nitric oxide and the release of IL-6 in LPS-induced murine macrophage cell RAW264.7. Additionally, IC_50_ values of **1**–**6**, **8**, and **9** on the release of TNF-α in murine macrophage cell RAW264.7 were close to their cytotoxic concentration, and therefore the decrease in TNF-α production may be an attribute of cytotoxity of analyzed compounds. It was the first time to systematically study anti-inflammatory activities of this kind of natural product. Structure-activity relationship studies revealed that the existence of the free carboxyl group in the structure of surfactin isomer was crucial as to the anti-inflammatory activities. So, it was important to choose an appropriate isolation procedure in the study of natural products, since inappropriate purification methods occasionally brought about artifacts and decreased bioactivities [16]. These findings will be very helpful for the development of this novel kind of natural product as new anti-inflammatory agents and potential immunosuppressive agents.

## Figures and Tables

**Table 1:** Pseudo-molecular ions and main product ions obtained from the positive and negative ions ESI-MS^n^ (n = 1, 2, 3) analyses of pure surfactin isomers.

	Pseudo-molecular ions (*m/z*)		Main product ions (*m/z*)	
Compound	[M + H]^+^	[M − H]^−^	MS^2^	MS^3^
1	a1036	1034	1018, 937, 905, b671	653, 554, 441
2	a1022	1020	1004, 923, 891, b671	653, 554, 441
3	a1008	1006	990, 895, 877, b685	667, 554, 441
4	a1022	1020	1004, 909, 891, b685	667, 554, 441
5	a1022	1020	1004, 891, b685	667, 554, 441
6	a1036	1034	1018, 923, 905, b685	667, 554, 441
7	a1050	1048	1032, 937, b699	681, 568, 455
8	a1036	1034	1018, 923, 905, b685	667, 554, 441
9	a1050	1048	1032, 937, 919, b685	667, 554, 441

a means precursor ion in the ESI-MS^2^;

b means precursor ion in the ESI-MS^3^.

**Table 2:** Peak numbers, retention times, pseudo-molecular ions and main product ions detected by HPLC-ESI MS^n^ (n = 1, 2, 3) analysis of the fraction (061341-A9) derived from *Bacillus* sp.

		Pseudo-molecular ions (*m/z*)		Main product ions (*m/z*)	
No	*t_R_*/min	a[M + H]^+^	[M − H]^−^	MS^2^	MS^3^
1	6.04	1040	1038	1022, 909, b685	554, 441
2	6.51	1040	1038	1022, 909, b685	554, 441
3	7.02	994	992	976, 863, b685	-
4	7.44	1054	1052	1036, 923, 909, b685	441, 228
5	7.81	994	992	-	-
6	8.22	1008	1006	990, 895, 877, b685	667, 554, 441
7	9.25	994	992	976, 895, 671	653, 554, 441
8	10.54	1022	1020	1004, 909, 891, b685	667, 554, 441
9	11.51	1022	1020	1004, 891, b685	667, 554, 441
10	12.07	1008	1006	990, 909, b671	554, 441
11	12.53	994	992	-	-
12	12.63	1022	1020	1004, 909, 891, b671	-
13	13.39	1036	1034	1018, 923, 905, b685	667, 554, 441
14	14.15	1022	1020	1004, 891, b685	-
15	14.80	1036	1034	1018, 923, b685	-
16	15.36	1022	1020	1004, 923, 891, b671	653, 554, 441
17	16.34	1022	1020	-	-
18	16.34	1050	1048	1032, 937, b685	667, 572, 395
19	17.75	1050	1048	1032, 937, 891, b685	667, 554, 441
20	18.21	1036	1034	1018, 923, 905, b685	667, 554, 441

a means precursor ion in the ESI-MS^2^;

b means precursor ion in the ESI-MS^3^.

**Table 3:** Inhibitory effects of surfactin isomers (**1**–**9**) on NO production induced by LPS in RAW264.7 cells.

Compound	Inhibitory rate on the NO production Concentration (μM)				IC_50_ (μM)
	30	10	3	1	
1	101.53 ± 0.70	99.02 ± 1.05	38.04 ± 4.01	19.95 ± 1.40	4.37
2	101.78 ± 1.05	99.79 ± 0.35	78.44 ± 4.91	34.08 ± 1.05	1.72
3	103.06 ± 3.51	84.39 ± 7.53	38.79 ± 0.70	4.83 ± 3.86	4.72
4	103.80 ± 0.35	101.32 ± 2.45	65.56 ± 4.21	24.66 ± 4.56	2.24
5	101.78 ± 0.35	101.61 ± 2.10	61.34 ± 1.05	18.46 ± 2.10	2.47
6	103.55 ± 0.87	102.32 ± 2.45	70.76 ± 2.45	30.36 ± 0.70	1.97
7	35.07 ± 5.96	31.10 ± 6.66	31.85 ± 8.41	21.68 ± 0.35	>30
8	103.06 ± 0.70	100.73 ± 2.10	57.38 ± 2.45	20.94 ± 1.40	2.59
9	102.27 ± 0.35	98.39 ± 1.75	67.29 ± 4.56	19.70 ± 0.35	2.27
ΔHydrocortisone					64.34

* Values are means of four experiments;

Δ Hydrocortisone was used as a positive control.

**Table 4:** Inhibitory effects of surfactin isomers (**1**–**9**) on IL-6 and TNF-α production induced by LPS in RAW264.7 cells.

	Compounds (IC_50_, μM)									
	1	2	3	4	5	6	7	8	9	ΔHydrocortisone
**IL-6**	5.90	1.16	6.64	4.95	1.79	5.48	>30	4.54	5.71	63.86
**TNF-α**	29.29	12.85	20.19	13.29	15.30	12.61	>30	18.31	20.86	85.64

* Values are means of four experiments;

Δ Hydrocortisone was used as a positive control.
